# An open-source neurodynamic model of the lower urinary tract

**DOI:** 10.1098/rsos.242062

**Published:** 2025-10-08

**Authors:** Elliot Lister, Aidan McConnell-Trevillion, Milad Jabbari, Abbas Erfanian, Kianoush Nazarpour

**Affiliations:** ^1^ School of Informatics, The University of Edinburgh, Edinburgh, UK; ^2^ Iran University of Science and Technology, Tehran, Iran

**Keywords:** *In silico* medicine, open-source model, bladder model, kidney function, personalized medicine, lower urinary tract, computational modelling, urodynamics, neural control, overactive bladder

## Abstract

Lower urinary tract symptoms affect a significant proportion of the population. *In silico* medicine can help understand these conditions and develop treatments. However, many of the current lower urinary tract computational models are closed source, too deterministic and do not allow for simple use of modelling neural intervention. An open-source Python-based model was developed to simulate bladder, sphincter and kidney dynamics using normalized neural signals to predict pressure and volume. The model was verified against animal bladder data from adult male Wistar rats, assessed for noise sensitivity and evaluated against known physiological factors. The animal data comparison yielded a significantly more similar pattern than existing models, with a correlation coefficient of *r* = 0.93 (*p* < 0.001). All physiological factors were within bounds, and the model remained stable with noise under the described boundaries. The proposed model advances the field of computational medicine by providing an open-source model for researchers and developers. It improves upon existing models by being accessible, including a built-in neural model that better replicates smooth bladder filling results, and incorporating a novel kidney function that alters bladder function by time of day in line with circadian rhythm. Future applications include personalized medicine, treating lower urinary tract symptoms with *in silico* models and adaptive neural interventions.

## 1. Introduction

Lower urinary tract (LUT) symptoms are a diverse and highly prevalent set of conditions known to affect over 60% of adults by the age of 40 [1]. Overactive bladder (OAB), a type of LUT symptom [2], is particularly widespread—with a reported prevalence of 10.7% of the global population [3] and 12% in the UK alone [4]. OAB is typically characterized by urinary urgency, frequency and urge incontinence [4] and can impart significant impacts on quality of life, causing social embarrassment, sleep disruption and anxiety [5]. Despite the availability of effective treatments, such as bladder retraining and pharmaceutical intervention [6], limited knowledge about the underlying aetiology remains a significant barrier to the development of more efficient solutions with fewer side effects. This research gap may be addressed by the development of computational models of the LUT. Computational approaches to LUT research offer a number of benefits. Short-term benefits of these models include increased reproducibility, which allows researchers to share and validate their findings. Additionally, these models can contribute to a reduction in animal testing, promoting more ethical and efficient research practices. In the long term, clinicians and researchers may utilize simulated organs to integrate individual patient characteristics and unique disease states. This personalized approach enables the prediction of treatment outcomes under different scenarios, guiding the selection of the most effective treatment strategy. By minimizing time spent trialling ineffective treatment plans, valuable time and resources can be conserved. This benefit is particularly enticing given the stark economic cost of OAB, with £840 million spent annually in the UK on the treatment and management of the condition [7]. Moreover, models play a considerable role in the design and optimization of novel medical devices. Despite the clear benefits of a computational approach, present attempts at capturing the system fall short on several fronts.

A robust model of the LUT requires several key attributes. Firstly, the capacity to integrate external neural inputs is essential for capturing complex interactions with the nervous system. Secondly, a smooth neural model representation is essential to avoid unrealistic action potentials. Thirdly, incorporating variable kidney function is fundamental for preventing oversimplified and deterministic outcomes. Lastly, code accessibility is necessary to facilitate model sharing, validation, and broader application within the scientific community.

Current LUT models, see table 1, use unrealistic representations of biological processes, such as treating urine inflow from the kidneys as a constant rate. These assumptions may lead the model to deviate from physiological observations, thereby impeding its ability to accurately represent LUT behaviour. Moreover, some models suffer from problems related to reproducibility. The original papers may not provide enough detail for accurate replication, thereby creating obstacles for other researchers to effectively utilize and advance these models, consequently impeding scientific progress. While open-source LUT models are now becoming available [14], they lack the novel features discussed in this paper. Furthermore, to the best of our knowledge, no other open-source models provide such ease of use for the simulation of bladders and the LUT, meaning our model is more accessible and user-friendly for researchers, particularly those in clinical settings or other fields who may not have a strong background in computational modelling.

**Table 1.** Comparison of existing models and their parameters.

model	inputs	neural model	kidney function	outputs	code availability
our model	neural units for detrusor activation, detrusor inhibition and sphincter activation	piecewise Gaussian	stochastic	pressure and volume by unit time	open access
Valentini, Besson and Nelson (VBN) [8]	gender, initial bladder volume, detrusor and sphincter activity, and urethral properties	step function with decay components	N/A	flow rates and bladder pressure over time	upon request
Hosein & Griffiths [9]	N/A	threshold-based, firing-rate model with afferent feedback	constant	pressure and volume by unit time	upon request, but in legacy Pascal
Fletcher *et al*. [10]	neural units for bladder musculature and urethral relaxation	exponential	N/A	pressure and volume by unit time	no
Paya *et al.* [11]	N/A	neural regulator system	constant	pressure and volume by unit time	no
Bastiaanssen *et al.* [12]	neural units for detrusor activation, detrusor inhibition and sphincter activation	manually tuned, firing-rate neural network [13]	constant	pressure and volume by unit time	no

This paper aims to overcome these limitations by introducing a novel and rigorously validated computational model of the LUT. Our model incorporates several significant modifications. Firstly, it features a dynamic urine inflow that reflects the body’s natural fluctuations in urine production throughout the day, mimicking natural physiological patterns for a more accurate representation of bladder filling. Secondly, the model incorporates elements of randomness to account for the inherent variability present in biological systems. The inclusion of stochasticity in the model enhances its realism, ensuring that it accurately represents the full range of bladder behaviour while operating within safe limits. Thirdly, we use Gaussian functions to model neural inputs from the brain, resulting in a more gradual rise in bladder pressure compared to existing models that utilize step-like functions. Finally, recognizing the importance of collaboration and knowledge sharing in scientific progress, we have made the code associated with this model open access for other researchers to utilize and build upon.

**Figure 1.**
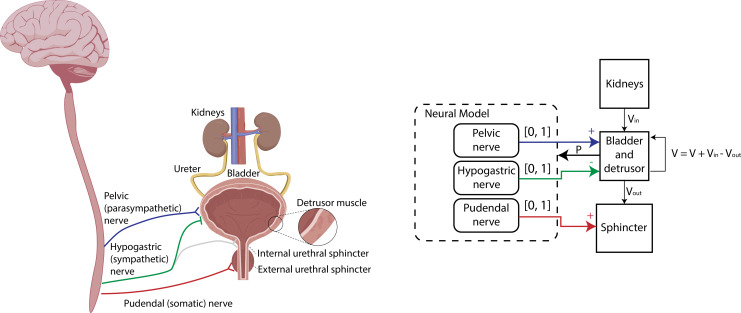
Left: anatomical diagram of the lower urinary tract and kidney for the proposed model, highlighting the detrusor muscle, urethral sphincters and their innervation. Right: computational analogue of the LUT system.

Figure 1 illustrates the biological system (left) and its computational analogue (right). Three normalized neural inputs modulate the detrusor and sphincter muscles, thereby controlling outflow rate. Urine inflow, modelled independently by a kidney function, determines bladder filling. A feedback loop incorporates bladder pressure to dynamically update the neural model.

This comprehensive and accessible model paves the way for the development and testing of in silico interventions. Through offering a more realistic portrayal of bladder function, our model can have a significant impact in multiple domains.

## 2. Method

### 2.1. Core bladder function

Our model, inspired by the framework developed by Bastiaanssen *et al.* [12], is a lumped model [15] representing the bladder as a single compartment to simulate filling and emptying. It uses three normalized inputs to represent the neural control of the bladder and sphincter: $\omega_{e}^{*}$ (excitatory input of the detrusor muscle), $\omega_{i}^{*}$ (inhibitory input of the detrusor muscle) and $\omega_{s}^{*}$ (excitatory input of the sphincter), corresponding to the pelvic, hypogastric and pudendal nerves, respectively. The model focuses on three state variables, namely,

— $V_{B}$: The volume within the bladder,
— $f_{aD}^{*}$: The normalized activation of the detrusor muscle, and
— $f_{aS}^{*}$: The normalized activation of the sphincter muscle.

Each state variable is modelled and updated by a differential equation, where the neural activations make use of time constants $\tau_{D}$ and $\tau_{S}$. We refer to the functions that model each equation as $f_{1}$, $f_{2}$ and $f_{3}$, respectively


(2.1) $$\begin{array}{lcr} & \begin{array}{rl} \frac{\text{d}V_{B}}{\text{d}t} & =f_{1}(f_{aD}^{*},V_{B},Q)=Q_{\text{in}}-Q\\ \tau_{D}\frac{\text{d}f_{aD}^{*}}{\text{d}t} & =f_{2}(f_{aD}^{*},\omega_{e}^{*},\omega_{i}^{*})=\omega_{e}^{*}-f_{aD}^{*}-\omega_{i}^{*}f_{aD}^{*}\\ \tau_{S}\frac{\text{d}f_{aS}^{*}}{\text{d}t} & =f_{3}(f_{aS}^{*},\omega_{s}^{*})=\omega_{s}^{*}-f_{aS}^{*}. \end{array} & \end{array}$$

Here, $Q_{\text{in}}$ is the urine inflow determined by the kidney model. Q is the urine outflow, which is a function of the urethral opening radius $r_{U}$, determined using a mapping function $f_{\mathrm{map}}$ based on $V_{B}$, $f_{aD}^{*}$, and $f_{aS}^{*}$. The root of the function $f_{0}(V_{B},\;f_{aD}^{*},\;f_{aS}^{*},\;r_{U})$ is found using the bisection method, implemented with the SciPy package [16]. If the bisection method fails, $r_{U}$ defaults to zero, assuming the urethra remains closed.

Muscle tension in the sphincter and detrusor includes both active and passive components. Passive tension is modelled by an exponential relationship dependent on the urethral radius $r_{U}$


(2.2) $$p_{\text{pas}S}=p_{0S}\cdot {\left(\frac{p_{\text{nomS}}}{p_{0S}}\right)}^{\frac{r_{U}}{r_{0S}}}.$$

Active pressure within the urethra depends on the muscle tension in the sphincter wall ($\sigma_{S}$) and the curvature of the sphincter. Given the assumption that the sphincter muscle can be modelled as a cylinder, it exhibits unidirectional bending. Hence, the active pressure can be represented by the equation


(2.3) $$\begin{array}{lcr} & \begin{array}{rl} p_{\text{act}S} & =\int_{r_{\text{in}S}}^{r_{\text{out}S}}\frac{\sigma_{\text{act}S}}{r}dr\\ & =\sigma_{\text{act}S}\ln\left(\frac{r_{\text{out}S}}{r_{\text{in}S}}\right). \end{array} & \end{array}$$

The active tensile stress $\sigma_{nom\_actS}$ is calculated based on the normalized activation $f_{aS}^{*}$, isometric stress $\sigma_{\mathrm{isoS}}$ and the force–velocity relationship $\sigma_{uS}(u_{S}^{*})$


(2.4) $$\sigma_{nom\_actS}=f_{aS}^{*}\cdot \sigma_{isoS}\cdot \sigma_{uS}\left(u_{S}^{*}\right)\cdot \sigma_{lS}^{*}\left(l_{S}\right).$$

The contraction velocity $u_{S}$ is determined by the rate of change of the sphincter radius $r_{S}$


(2.5) $$\begin{array}{lcr} & \begin{array}{rl} u_{S} & =\frac{-dl_{S}/l_{\mathrm{opt}S}}{dt}\\ & =-\frac{1}{r_{\mathrm{opt}S}}\frac{dr_{S}}{dt}. \end{array} & \end{array}$$

The rate of change of $r_{S}$ is given by


(2.6) $$\begin{array}{lcr} & \begin{array}{rl} \frac{dr_{S}}{dt} & =\frac{r_{U}}{2}\left(\frac{1}{r_{\text{out}S}}+\frac{1}{r_{\text{in}S}}\right)\frac{dr_{U}}{dt}. \end{array} & \end{array}$$

Thus, the contraction velocity of the sphincter muscle is


(2.7) $$\begin{array}{lcr} & u_{S}=-\frac{r_{U}}{2r_{\mathrm{opt}S}}\left(\frac{1}{r_{\text{out}S}}+\frac{1}{r_{\text{in}S}}\right)\frac{dr_{U}}{dt}. & \end{array}$$

Hence, the pressure in the sphincter muscle can be given as


(2.8) $$\begin{array}{lcr} & p_{S}=p_{\text{act}S}+p_{\text{pas}S}. & \end{array}$$

Bastiaanssen *et al.* [12] derived a relation between the urethral radius ($r_{U}$) to the square rate of urine flow ($Q^{2}$) as


(2.9) $$\begin{array}{lcr} & Q^{2}=\frac{p_{S}(r_{U})A_{U}^{2}(r_{U})}{\frac{\rho }{2}(1-A_{U}^{2}(r_{U})/A_{T}^{2}(r_{U}))+(R(r_{U})A_{U}^{2}(r_{U}))}, & \end{array}$$

where $A_{U}$ is the cross-sectional area occupied by urine. The detrusor muscle pressure calculation follows a similar approach, with the contraction speed $u_{D}$ given by


(2.10) $$\begin{array}{lcr} & \begin{array}{rl} u_{D} & =-\frac{1}{r_{\mathrm{opt}D}}\frac{dr_{D}}{dt}\\ & =-\frac{1}{8\pi r_{\mathrm{opt}D}}\left(\frac{1}{r_{\text{out}D}}+\frac{1}{r_{\text{in}D}}\right)(Q_{\mathrm{in}}-Q). \end{array} & \end{array}$$

Assuming $Q_{\mathrm{in}}=0$, the detrusor muscle tensile stress includes elastic and viscoelastic elements of the bladder wall. The objective function ($f_{0}$) calculates the pressure difference between the bladder neck and the urethra entrance, considering factors like bladder volume ($V_{B}$), muscle contraction strength ($f_{aD}^{*}$), sphincter resistance ($f_{aS}^{*}$) and urethral radius ($r_{U}$). The bladder volume ($V_{B}$) is updated based on the calculated net flow ($f_{1}$) through the use of the following equation:


(2.11) $$\begin{array}{lcr} & V_{B}(t+\Delta t)=V_{B}(t)+f_{1}(t)\Delta t. & \end{array}$$

For detailed derivations and a thorough explanation of the underlying formulas, readers are referred to the original paper [12].

### 2.2. Neural model

The neural control model used with the bladder model [13] could not be replicated due to insufficient data and descriptions of fitting techniques. Therefore, we developed a new model using piecewise Gaussian functions to simulate the neural inputs. Each input is modelled using a left-skewed Gaussian function, where the slope is smooth until the point of voiding, at which the inputs are fixed as constants. The bladder excitatory ($\omega_{e}^{*}$) and inhibitory ($\omega_{i}^{*}$) inputs are functions of the bladder volume ($V_{B}$). These inputs provide feedback based on the current volume of the bladder. The functions are defined as follows:


(2.12) $$\begin{array}{lcr} & \begin{array}{r} \omega_{e}^{*}(V_{B})=\left\{ \begin{array}{cc} A_{e}\exp\left(-\frac{(V_{B}-\mu_{e})2}{2\sigma_{e}2}\right) & \text{if }V_{B}<V_{\text{void}}\\ \text{constant} & \text{if }V_{B}\geq V_{\text{void}} \end{array}\right. \end{array} & \end{array}$$

(2.13) $$\begin{array}{lcr} & \begin{array}{r} \omega_{i}^{*}(V_{B})=\left\{ \begin{array}{cc} A_{i}\exp\left(-\frac{(V_{B}-\mu_{i})2}{2\sigma_{i}2}\right) & \text{if }V_{B}<V_{\text{void}}\\ \text{constant} & \text{if }V_{B}\geq V_{\text{void}}. \end{array}\right. \end{array} & \end{array}$$

Here, $A_{e}$ and $A_{i}$ are the amplitudes, $\mu_{e}$ and $\mu_{i}$ are the means and $\sigma_{e}$ and $\sigma_{i}$ are the standard deviations of the Gaussian functions. $V_{\text{void}}$ is the bladder volume at which voiding occurs, and beyond this point, the inputs are fixed as constants. The sphincter muscle excitatory input ($\omega_{S}^{*}$) is a function of the urethral radius ($r_{U}$). This input provides feedback based on the current radius of the urethra. The function is defined as follows:


(2.14) $$\begin{array}{r} \omega_{s}^{*}(r_{U})=\left\{ \begin{array}{ll} A_{s}\exp\left(-\frac{(r_{U}-\mu_{s})2}{2\sigma_{s}2}\right) & \text{if }V_{B}<V_{\text{void}}\\ \text{constant} & \text{if }V_{B}\geq V_{\text{void}}. \end{array}\right. \end{array}$$

Similarly, $A_{s}$ is the amplitude, $\mu_{s}$ is the mean and $\sigma_{s}$ is the standard deviation of the Gaussian function. This piecewise Gaussian approach allows for smooth transitions in neural inputs until the point of voiding, ensuring realistic simulation of bladder and sphincter muscle control.

### 2.3. Kidney function

Modelling the state of kidney outflow for an individual is challenging because of factors such as liquid intake, food consumption, medications, other medical conditions, age and perspiratory behaviour [17]. Given the number of parameters involved, it would not be feasible to implement or validate a kidney model relying on these factors. However, there is one factor that universally affects bladders: time. Although the production of urine in the kidneys does not change intensely throughout the day, it has been shown that numerous functions of the kidney, including urine production, vary with circadian rhythm [18,19]. Based on this information, the inflow ($Q_{\mathrm{in}}$) has been represented mathematically as a sinusoidal function.


(2.15) $$Q_{in}=(Q_{\text{max}}\sin(ft-\phi )+C)\cdot n,$$

where $Q_{\text{max}}$ is the max inflow, f is the urinary frequency, t the simulation time, ϕ the phase shift, C a constant term and n the uniform noise. After each minute (or chosen time step), a random scalar (n) uniformly distributed between −1 and 1 is multiplied by the volume obtained from the sine function. This method preserves the overall circadian pattern while introducing controlled variability. As insufficient public data exist on per minute urine production, the function was fit to align with an average inflow that experiences fluctuations over a 24 h cycle within ranges of typical urine production. With that in mind, the numbers provided can be adjusted and personalized, thereby enhancing the model’s compatibility with users. Using this function creates peaks in urine production during the afternoon and weaker inflow during the early morning, aligning with what is reported in the literature [18]. Despite its increased complexity, this model of urine production can still be adjusted with just a few parameters if there is a significant difference between a patient’s flow rates and the current model fit.

### 2.4. Code availability

The code for implementing the bladder model, neural control model and kidney function can be accessed via the GitHub repository [20]. Access to the repository is open for researchers and developers who are interested in reviewing, replicating and building on the work presented in this study. The repository provides detailed documentation and examples, which should support users in understanding and utilizing the content.

### 2.5. Validation

**Figure 2.**
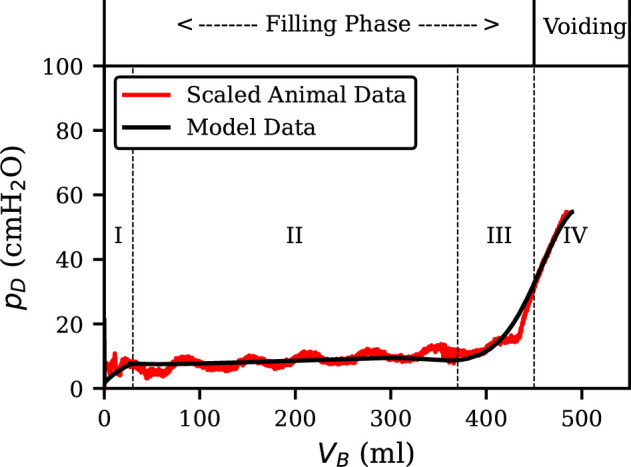
Pressure–volume relationship for scaled animal data and the human LUT model. The relationship aligns with an ideal human cystometrogram, as described in the literature [22], with distinct phases: an initial pressure rise (phase I), bladder compliance (phase II) and gradual pressure increase with voiding (phases III–IV). The scaled animal data and model outputs are in strong agreement, as evidenced by a Pearson correlation coefficient of *r* = 0.93 (*p* < 0.001).

Validating a biophysical LUT model is inherently challenging due to the invasive nature of acquiring high-quality clinical data on bladder pressure, volume and voiding dynamics. Ethical constraints limit experiments on healthy individuals, and variations in anatomy and physiology among patients complicate direct comparisons between model predictions and clinical observations. Additionally, privacy laws often restrict access to human data, hindering reproducibility. To address these challenges, we have validated model components using data from animal studies and published physiological ranges of human data. This involved comparing model outputs to established limits, reproducing published results and analysing model behaviour under known physiological perturbations. Utilizing data previously obtained from experiments on adult male Wistar rats [21], a two-step validation was employed to assess the LUT model’s ability to represent real-world physiology. The validation dataset is provided as a MATLAB file (RealBladderData.mat) in the GitHub repository. The raw data include time-series vectors of bladder pressure and infused, voided and residual volumes. As detailed in the provided Jupyter Notebook (figures.ipynb), these require pre-processing to align the signals onto a common time base for analysis. This animal data were used for validation purposes only and not for model fitting or parameter tuning. First, min–max scaling was applied to the animal data for visual comparison in figure 2 only, to bring it into a range comparable with the predictions of the human model. This allowed for a visual overlay of the scaled animal data onto the model’s outputs. This qualitative comparison provided valuable insights into the similarity between the model’s behaviour and the actual observations from the animal experiments. Secondly, a quantitative analysis using Pearson correlation was conducted. In this case, the ratio of pressure to volume for the non-scaled animal data was compared with the corresponding outputs from the human model.

## 3. Results

This section reports the core outputs and dynamics of the proposed bladder model. We explore the pressure–volume relationship and compare our simulations to experimental animal data. Additionally, we examine key physiological metrics such as voiding durations and filling times. This is followed by an assessment of the model’s robustness to stochastic variability in the kidney inflow. The section concludes with a systematic sensitivity analysis to quantify the influence of the novel model parameters on key outputs and a state-space analysis to characterize the model’s dynamic cycle.

### 3.1. Model behaviour

**Figure 3.**
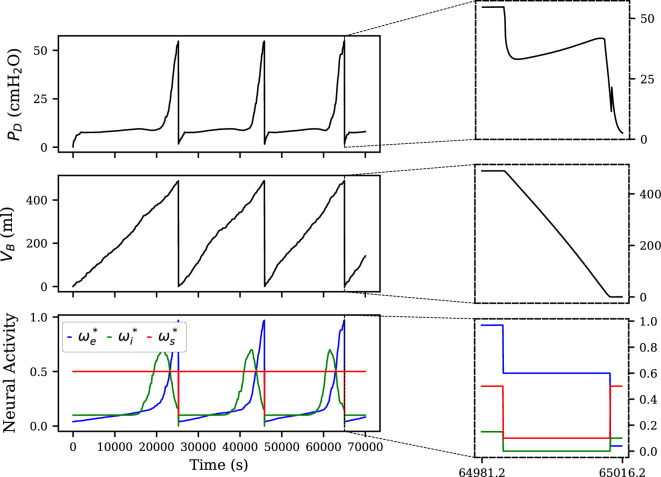
Time-series plots illustrating the dynamic behaviour of the model, including bladder pressure, volume and neural activity. A zoomed-in view of the voiding phase is also provided to highlight the rapid changes in these variables during micturition. $V_{B}$: bladder volume, $P_{D}$: detrusor pressure, $\omega_{e}^{*}$: excitatory bladder neural input, $\omega_{i}^{*}$: inhibitory bladder neural input, $\omega_{s}^{*}$: sphincter neural input.

Figure 3 illustrates the dynamic behaviour of the model, showcasing the time-series evolution of bladder pressure, volume and neural activity. The plot demonstrates the expected cyclic nature of bladder function, alternating between storage and voiding phases. During the storage phase, bladder volume gradually increases in a stochastic manner, reflecting the random inflow from the kidneys. As the bladder fills, neural activity gradually increases, modelled by a piecewise Gaussian function. To simulate the rapid transitions during the voiding phase, a simplified method was utilized. In this method, the neural units remain constant during voiding, as depicted in figure 3, leading to a stepwise alteration in neural activity. This simplification allows for easier manipulation of voiding dynamics, enabling the exploration of various scenarios and the tuning of model behaviour (e.g. voiding speed). For example, increasing the sphincter neural input ($\omega_{s}^{*}$) from 0.1 to 0.25 during voiding can significantly prolong voiding time, from 27.1 to 308.6 s. Conversely, decreasing this input to 0.01 can shorten voiding time to 25.4 s. Similarly, increasing the excitatory input to the bladder ($\omega_{e}^{*}$) from 0.6 to 1.0 can, as expected, reduce voiding time to 20.2 s due to the increased bladder pressure. These simulations highlight the model’s sensitivity to parameter changes and its ability to capture a range of physiological behaviours. While careful consideration should be given when modifying these values to avoid unintended consequences, this flexibility is a key advantage of the model, allowing for the exploration of various neurotherapeutic strategies and the development of innovative neuromodulation techniques. In the subsequent subsections, the neural model parameters will be reset to their original values, and further tests will be performed to validate the LUT model.

### 3.2. Pressure–volume relationship

In contrast to Bastiaanssen’s neural model [13], our model results in a slower increase in pressure. This modification in the neural weights’ interaction with the model leads to a less pronounced slope in the pressure–volume relationship. To facilitate comparison with literature, the detrusor pressure has been converted to units of ${\text{cmH}}_{2}\text{O}$. The pressure–volume plot in figure 2 exhibits a close resemblance to an ideal cystometrogram of a typical adult human female [22], characterized by an initial rise in pressure during phase I, subsequent bladder compliance in phase II, and concluding with a gradual pressure increase and voiding during phases III–IV.

The scaled animal data in figure 2 exhibited high similarity when overlaid on the human model outputs. This suggests the model effectively captures the general trends observed in the animal experiments. Further strengthening this validation, the Pearson correlation coefficient between the non-scaled animal data and the model outputs yielded a statistically significant value of r=0.93 (p<0.001). This high correlation value highlights a strong positive relationship between the model’s predictions and the *in vivo* data, providing further confidence in the model’s accuracy. However, the animal data do demonstrate fluctuations during the compliance phase of storage. This could be due to muscle spasms or noise produced in the recording of the small pressure values.

During phase II of figure 2, the bladder exhibits compliance as it stretches with minimal increase in detrusor pressure. For a bladder to be considered normal, its compliance should be over $40\;\text{ml}/{\text{cmH}}_{2}\text{O}$ [23]. Given the non-deterministic nature of each voiding cycle, a simulation was performed to assess compliance in every filling cycle. The findings revealed that compliance surpassed $40\;\text{ml}/{\text{cmH}}_{2}\text{O}$ across all cycles, indicating a healthy bladder.

### 3.3. Voiding dynamics

**Figure 4.**
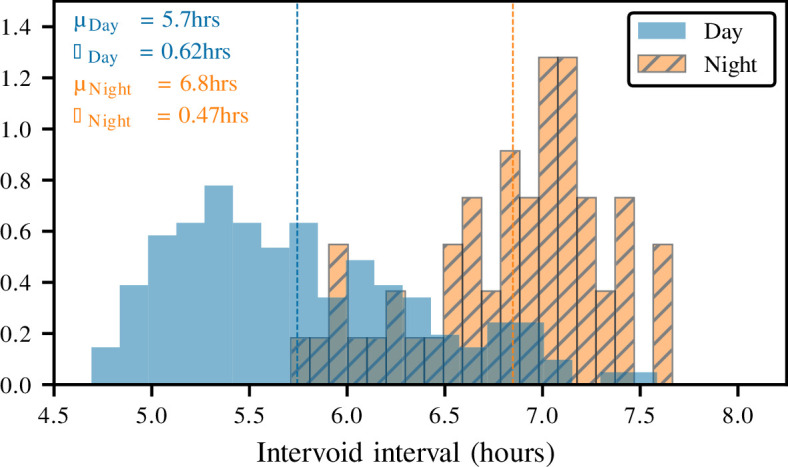
Distribution of intervoid intervals. The histogram displays the distribution of times between voiding events. During the day, the model demonstrates a lower mean intervoid interval, indicating more frequent voiding. Conversely, during the night, the model shows less frequent voiding. This behaviour is attributed to the stochastic kidney model integrated into the LUT framework, effectively simulating the natural variations in kidney function over a 24 h period.

The stochastic nature of the neural model results in a variable interval between voids, requiring the model to run over 50 days to extract the interval from each void cycle. For the purposes of grouping the intervals, it was assumed that night falls between 10 pm and 6 am; however, this will vary depending on the sleep cycle of the individual. Figure 4 displays the distribution of time intervals between voiding events. While the daytime voiding pattern aligns with the expected 5−7 h interval, the literature suggests that individuals with lower bladder capacities may experience more frequent voiding, approximately every 2−3 h [24]. This increased frequency is likely due to the reduced bladder capacity, which necessitates more frequent voiding events to maintain continence. Therefore, the observed voiding pattern aligns with a lower bladder capacity. On the other hand, the night voids seem to be shorter than expected, with the model experiencing voiding on some night. Although this is not uncommon, it could indicate that the filling model needs to be revised beyond a singular sine curve.

Alongside the assessment of the intervoid interval, the investigation also explored the distribution of voiding durations over the course of the 50 day simulation. The voiding events exhibited a duration of 27.3±0.05s, demonstrating that voiding durations are independent of the preceding bladder filling time. The literature suggests an average voiding duration of 21±13s [25]. While the value in this simulation exceeded the expected duration slightly, it remained within the range established by the literature. The observed difference suggests that modifications to the neural control model, as explored in §3.1, may be required for improved synchronization with the voiding dynamics depicted in the current model. The LUT model itself, however, appears to be functioning within a physiologically relevant range.

### 3.4. Model robustness to stochastic inflow

**Figure 5.**
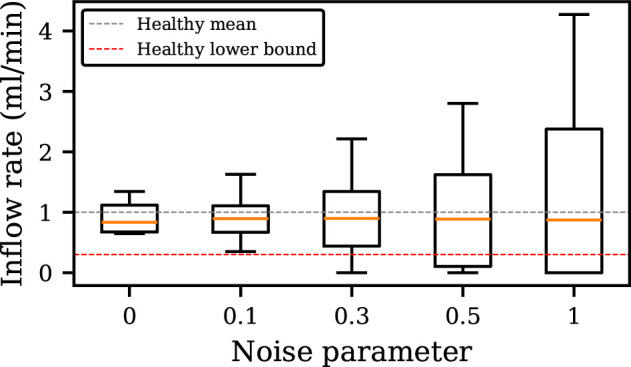
Box plot illustrating the distribution of filling rates under different noise parameter values, with reference lines showing the lower bound of 0.3 ml min^−1^ (dashed red) for a healthy filling rate (below which oliguria may be present) and the mean healthy inflow of 1 ml min^−1^ (grey).

To evaluate the model’s robustness to random fluctuations in the kidney inflow rate, simulations were conducted with varying noise parameter values. The resulting filling rates were analysed and visualized in a box plot (figure 5). The box plot demonstrates a clear relationship between the noise parameter and the variability of filling rates. As the noise parameter increases, the interquartile range of the filling rates expands, indicating a wider distribution of values. This suggests that the model’s output becomes more sensitive to random fluctuations as the noise level rises. The physiologically relevant range for filling rates is typically defined by a minimum inflow of around 0.3 ml min^−1^, below which oliguria may be indicated [26], and a maximum inflow of around 10 ml min^−1^ [27], with an average inflow of approximately 1 ml min^−1^ [27]. When noise parameters are ≤1, the average filling rates fall within this physiological range, with minimal outliers. This shows that the model maintains robustness under these conditions. However, when the noise parameter exceeds 1, the distribution of filling rates becomes more skewed, with a higher frequency of extreme values. When the randomly generated noise component is sufficiently negative, the net inflow drops below zero. To maintain biophysical accuracy, the model imposes a minimum inflow of $Q_{\text{in}}=0$. This prevents unrealistic negative inflows from the kidneys, which are not physiologically possible. Additionally, as the frequency of zero inflows increases, the average inflow rate decreases, leading to longer intervoid intervals. This can result in less frequent voiding, which may not accurately reflect physiological behaviour. While lower noise reduces this effect, it comes at the cost of increased model determinism. So, even though the model occasionally has periods of non-filling at higher noise values (0.5≤n≤1), these instances are not a major concern because they occur infrequently enough that the average remains within a safe level. Hence, to ensure model robustness and maintain biophysical accuracy, it is recommended to keep the noise parameter within the range of 0–1. If significantly more noise is required, the noise generation method itself should be reconsidered rather than solely adjusting the parameter.

The impact of noise on other model parameters was further explored through an analysis of the distribution of parameter values in both voiding and non-voiding phases. This distinction results from the fact that parameters such as the outflow rate, Q, behave differently in each of these phases; a combined analysis would fail to observe these differences and confirm stability. The distribution of model values during voiding (left, orange) and non-voiding (right, black) phases is illustrated in electronic supplementary material, figure S1. The results show the model is robust to random inflow rate fluctuations, as the noise parameter exhibits an insignificant effect on the distribution of other parameters.

### 3.5. Sensitivity analysis

To systematically quantify the influence of each novel parameter on model behaviour, a local one-at-a-time sensitivity analysis was performed by measuring the relative percentage change to the baseline model after perturbing each parameter. As these parameters lack empirically defined physiological ranges, their influence was assessed by varying each from its default value by ±5%. Three primary outputs were measured: mean intervoid interval, defined as the average time between the start of consecutive voiding events; mean bladder capacity, defined as the average bladder volume measured at the onset of each void; and mean voided volume, defined as the average total volume expelled during each voiding event.

**Figure 6.**
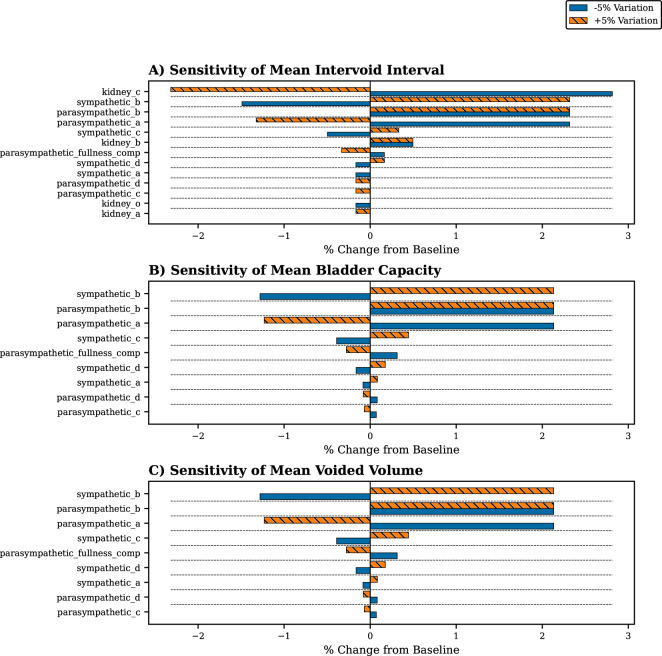
Sensitivity analysis of novel model parameters (see electronic supplementary material, table S1, for a complete list of parameters). Percentage change in three key model outputs in response to an individual ±5% perturbation of each novel parameter from its default value. The three panels correspond to the sensitivity of (A) mean intervoid interval, (B) mean bladder capacity and (C) mean voided volume. Orange/hatched bars represent the effect of a +5% parameter variation, while blue bars represent the effect of a −5% variation.

The sensitivity analysis was conducted on all new parameters introduced in the kidney and neural models. The parameters are named according to their respective subsystem (kidney, parasympathetic input, sympathetic input or somatic input) followed by a parameter name/identifier. A complete parameter list is provided in electronic supplementary material, table S1. The analysis revealed that several of these parameters had a negligible influence on the output metrics. Specifically, the parameters for the somatic pathway and the default activation values during voiding (..._w_voiding) had no significant impact on any of the three metrics. The low sensitivity of the somatic pathway is an expected outcome of the model’s state-based design for the neural controller. The transition from the Gaussian-controlled storage phase to the fixed-value voiding state is highly decisive; while a parameter perturbation may alter the smoothness of this transition on a micro-scale, the switch is sufficiently rapid that it does not produce a measurable change in the macroscopic outputs. Furthermore, while the kidney function parameters were influential on the intervoid interval, they did not affect the other voiding phase metrics. Therefore, to improve the clarity of the visualization in figure 6, all results for zero-variability parameters have been omitted, allowing for a clearer focus on the most influential relationships.

The results reveal a physiologically plausible separation of controls within the model. The model’s storage function is most influenced by the parameters of the kidney, with the intervoid interval being most sensitive to the mean inflow rate (kidney_c), followed by the neural parameters that define the point of peak sensitivity for the excitatory (parasympathetic_b) and inhibitory (sympathetic_b) systems. This finding is physiologically intuitive, as while urinary frequency is fundamentally constrained by the rate of bladder filling, it is also modulated by the neural control parameters that govern bladder sensitivity. In contrast, the voiding phase dynamics are primarily governed by the neural controllers. The bladder capacity and quantity of volume voided are most sensitive to the parameters that define the activation thresholds of these controllers (sympathetic_b and parasympathetic_b). This indicates that the precise timing of the neural switch from storage to voiding is the most critical factor in determining the model’s capacity and void quantities. The analysis also highlighted different sensitivity profiles. Most parameters exhibited an intuitive symmetrical response; for example, decreasing the mean kidney inflow (kidney_c) increased the intervoid interval, while increasing the inflow decreased it. In contrast, a few parameters showed an asymmetrical response. Notably, for parasympathetic_b, a perturbation of ±5% produced a similar positive change in the intervoid interval. This is an expected consequence of the squared term in the model’s Gaussian functions.

To further explore the model’s robustness beyond the local analysis, a wider parameter sweep of ±25% was also conducted, with the full results presented in electronic supplementary material, figure S2. This analysis confirmed that the outputs of the model remain relatively unchanged for most parameters, even when varied by up to ±25%. The kidney_c parameter remained the most pronounced influence on the intervoid interval. As expected, an inverse relationship is observed, where urine inflow is inversely proportional to the time between voids. However, the output variation increases at a rate less than that of the parameter itself, which indicates that the system remains stable. The analysis also identified a threshold for somatic_a, the parameter that represents the magnitude of the somatic input. If reduced significantly (≤ −15%), the model fails to reach the pressure threshold required for a void, leading to a state of continuous incontinence. This defines a clear boundary for the model’s normal operational range.

These findings collectively increase confidence in the model’s credibility as a reliable simulation tool for further investigation. This analysis indicates that the model exhibits robustness to parameter uncertainty, with most perturbations producing only minor, non-chaotic changes to the outputs. Moreover, the model’s key functions are predictably and logically controlled by the relevant subsystems.

### 3.6. Simulating clinical observations

Simulations were conducted to explore the model’s ability to reproduce trends observed in pathological conditions. OAB is often associated with detrusor sensitivity [28], which can result in symptoms such as increased frequency (leading to a decreased duration between voids) and decreased bladder capacity [29]. Bladder outlet obstruction (BOO) is often associated with increased urethral resistance, which can result in decreased urine flow rate [30]. Given the challenges associated with obtaining patient-specific parameter adjustments, this subsection will analyse the model’s ability to reproduce trends established within the existing literature. The hypotheses, along with all associated parameter modifications and the complete experimental Jupyter Notebook, are available on GitHub [20]. Specific parameter changes for each simulated condition are also detailed in electronic supplementary material, table S2.

*Hypothesis 1: Simulating OAB by increasing parasympathetic activation and decreasing sympathetic activation results in decreased intervoid interval.* Clinically, OAB is characterized by an increased frequency of urination, which corresponds to shorter intervals between voids [29]. To simulate trends characteristic of OAB, the neural circuit governing bladder function was modified to increase parasympathetic activation and decrease sympathetic inhibition across the bladder filling cycle. This change was chosen to represent the detrusor hypersensitivity commonly associated with the condition [28]. This modification effectively enhanced detrusor muscle excitation. Simulations demonstrated a reduction in the mean intervoid interval decreased by 31.04% (approximately 5.9–4.1 h) in this particular configuration.

*Hypothesis 2: Simulating OAB by increasing parasympathetic activation and decreasing sympathetic activation results in decreased maximum bladder capacity.* Using the same neural circuit modification as described in Hypothesis 1, the capacity was determined by identifying the maximum bladder volume across the simulation. The maximum bladder capacity decreased from 489.58 ml in the control to 334.89 ml in the modified model, a reduction of 31.60%. Together, these results align with the primary symptoms of OAB.

*Hypothesis 3: Simulating BOO by increasing urethral resistance results in decreased flow rate.* BOO is defined by an increased urethral resistance that impedes urination, leading to a decreased urine flow rate [30]. To simulate trends characteristic of BOO, the urethral resistance was modified by increasing the parameters governing urethral outflow resistance ($R_{1}$ and $R_{2}$ in the model). ‘$R_{1}$’ was increased from $3.0\times 10^{8}$ N to $15.0\times 10^{8}$ N, and ‘$R_{2}$’ was increased from $2.4\times 10^{8}$ N to $12.0\times 10^{8}$ N, a five-fold increase chosen to represent a significant physical obstruction. The maximum urine flow rate decreased from 21.1 ml s^−1^ in the control to 12.31 ml s^−1^ in the modified model (a reduction of 41.71%), indicating bladder obstruction.

### 3.7. State-space analysis

**Figure 7.**
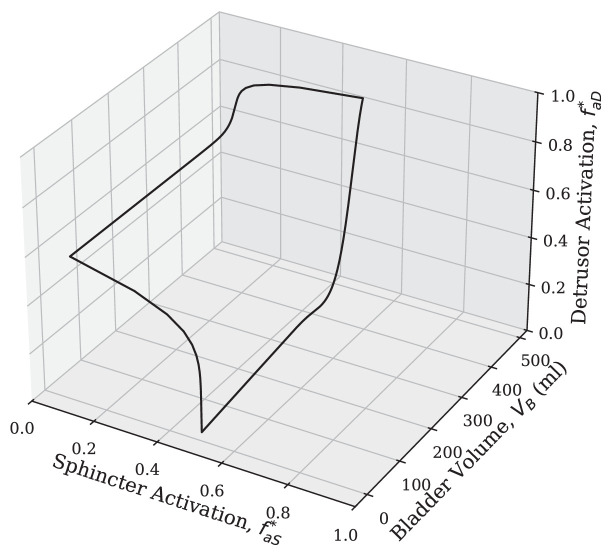
State-space trajectory of the model. A three-dimensional plot of the primary state variables—bladder volume ($V_{B}$), detrusor activation ($f_{aD}^{*}$) and sphincter activation ($f_{aS}^{*}$)—over a complete micturition cycle.

To characterize the dynamic behaviour of the model, a state-space analysis was performed. As previously noted, the model’s state is characterized by three variables: bladder volume ($V_{B}$), detrusor activation ($f_{aD}^{*}$) and sphincter activation ($f_{aS}^{*}$). The resulting trajectory of a full micturition cycle is presented in figure 7. The trajectory’s stable, continuous loop demonstrates the cyclical nature of bladder function. Two distinct phases are clearly delineated. The lower portion of the loop represents the filling phase, characterized by increasing bladder volume under conditions of low detrusor activity and high sphincter activation. In the upper portion, the plot illustrates the voiding phase, characterized by a rapid volumetric decline due to elevated detrusor activity and reduced sphincter activation. The continuous nature of the transitions between these phases is a direct consequence of the Gaussian-based neural control model.

## 4. Conclusion

This paper presents a novel approach for developing an open-source LUT model incorporating sinusoidal kidney function and noise injection. As detailed in table 1, the model offers the following key attributes: the availability to integrate external neural inputs, smooth neural model representation, variable kidney function and code accessibility. Our model is a lumped model, classifying the bladder as a single compartment to represent its storage and voiding functions. Building upon the framework initially established by Bastiaanssen *et al.* [12], this approach provides a high-level overview of LUT function, effectively balancing physiological realism with computational efficiency. Bastiaanssen’s original neural control model could not be replicated owing to the insufficient detail and training data provided in its description. Consequently, our model, although conceptually similar to their mechanical structure, exhibits substantial differences in its neural control representation, aiming for greater replicability and physiological accuracy in its input process. Many existing LUT models, particularly those focusing on the neural aspects, have constructed abstract representations of neural signals. For instance, Hosein & Griffiths [9] introduced a model where nerve activity was assumed to jump quickly between states of storage and voiding, based on two mutually inhibitory control regions. Similarly, van Duin *et al.* [31] explored various neural control configurations, modelling nodal behaviour with logistic functions. Paya *et al.* [11] also simplified neural control by modelling discrete systems that activate when the inputs exceed a threshold. The framework presented in this paper is consistent with prior research, similarly employing threshold-based activation of modular neural units. However, it distinguishes itself by using piecewise Gaussian functions to continuously modulate the neural inputs as a function of bladder fullness. This design results in a gradual, nonlinear change in detrusor activation throughout the filling cycle. The primary benefit of this approach is the model’s ability to produce a pressure–volume relationship that closely aligns with the low-pressure compliance phase of an ideal human cystometrogram, a feature not explicitly demonstrated by earlier models that focused primarily on the switch to voiding.

Additionally, our model presents a significant innovation: the incorporation of variable kidney function, which dynamically influences bladder function in accordance with the circadian rhythm. This contrasts with the typical approach of existing LUT models, which make use of a constant rate of urine inflow from the kidneys. By incorporating this dynamic, time-of-day-dependent urine inflow, our model offers a more accurate representation of bladder filling under natural physiological patterns. We also explicitly include elements of randomness or stochasticity to account for the inherent variability observed in biological systems, a feature often overlooked in more deterministic models. Therefore, the model accurately represents the dynamic filling rate and unpredictable behaviour of the biological system, as validated against established physiological ranges and metrics. The model demonstrated robustness by maintaining physiologically healthy filling rates across varying levels of noise within the recommended range. This highlights the resilience of the model to random fluctuations and its ability to provide accurate predictions under different conditions. By introducing random fluctuations, the model can better realistically simulate the natural variations in physiological processes. This feature can lead to a more accurate representation of bladder function and improve the predictive capabilities of the model. Furthermore, the model’s dynamic stability was confirmed through state-space analysis, which shows the current implementation maintains a stable, physiological micturition cycle, establishing a reliable baseline with its default neural controller. This improves user confidence in the current system while allowing for the flexible development of its neural control system to suit varied research needs. In addition to the model’s core validation, this paper explored the model’s ability to reproduce trends observed in pathological conditions. The simulations demonstrated that the model can capture key characteristics associated with altered bladder function, such as changes in voiding patterns, bladder capacity and urine flow rate. Although direct clinical validation is limited, the model’s ability to replicate these established trends provides further support for its physiological relevance and potential utility in studying LUT dysfunction.

Jaskowak *et al.* [15] highlighted *‘*five simplest rules*’* that serve as minimum requirements for reproducibility in computational models [15]. This work has prioritized these principles, which are clearly demonstrated in several key aspects of our work. Firstly, aligning with the recommendation to define the context clearly, the introduction specifies that this model’s intended use is to simulate the dynamics of the LUT in order to predict bladder pressure and volume. Second, in accordance with the use of contextually appropriate data, our model validates against animal data and compares outputs to human physiological ranges. Third, the paper discusses the model’s assumptions and limitations, with dedicated text detailing key limitations in the following paragraph. Fourth, the model is version controlled and archived using a public repository [20]. This directly addresses the low adoption of this practice, as shown by Jaskowak *et al.* [15], whose review found that 0% of the surveyed models utilized version control via online repositories. Finally, the model is thoroughly documented, with installation instructions, usage guidelines, parameter inputs and example outputs all readily available on GitHub, ensuring appropriate documentation. This focus on these five key aspects aims to make our model more accessible and user-friendly for researchers, including those in clinical settings or other fields who may not have extensive computational modelling expertise, thereby promoting wider adoption and validation.

While this model offers advancements, it is important to acknowledge its limitations. To enhance computational efficiency and ease of use, a simplified representation of neural input is utilized; a comprehensive model of the neural circuitry underlying micturition is not included. For example, while our neural model provides a smoother representation of neural inputs, it does not delve into the intricate single-neuron dynamics that models like McGee and Grill explore with linear integrate-and-fire neurons [32], or de Groat *et al.* with switching circuits based on periaqueductal gray and pontine micturition center (PAG-PMC) activity [33]. Our current neural model is primarily a neural regulator system that modulates detrusor and sphincter muscles, rather than a full simulation of central nervous system pathways. Furthermore, as a lumped model, the system currently lacks a spatial representation of the bladder and associated structures. Future development of the model could incorporate spatial elements, such as regional variations in muscle activity or fluid flow. Finally, statistical model validation currently relies on animal data, which may not fully capture the complexities of human bladder physiology. Therefore, further validation with human data would strengthen the model’s applicability to clinical settings and enhance its potential for personalized medicine applications.

This work contributes to the field of *in silico* medicine by providing a valuable tool for studying and understanding bladder function. The availability of an open-source bladder model serves research, education and clinical applications. In its present form, the model is well suited to analyse pathological conditions associated with physiological or structural changes to the bladder. These include prevalent, life-altering conditions such as urinary tract infection [34], interstitial cystitis [35] and obstructive urinary retention [36,37], all of which would benefit considerably from an open-source computational framework for further analysis. From the point of view of future work, there is considerable potential benefit to the integration of this model with a simulation of its neural control system. The research team has recently adapted a modified version of this system, which successfully captures the intricate neural interactions underpinning the micturition cycle [38] which they used to analyse the system-level effects of neuromodulation. Were the model outlined here to be integrated with this neural network, the resulting work could be used to analyse a number of open problems within the LUT pathology literature.

These include the as-of-yet unknown mechanism behind the therapeutic effects of transcutaneous tibial nerve stimulation [39] or an in-depth exploration of the system-level changes that occur as a result of overactive [40] or underactive bladder [41]. The therapeutic value of answering these questions should not be understated. As such, future work will aim to merge these two models to produce a detailed framework that will allow for both bladder-specific and system-level simulation of pathologies and potential solutions.

## Data Availability

This simulation framework is open-source and as such any code used in this publication is available on the research team GitHub [20]. The experimental data used for validation is also available in the repository. Supplementary material is available online [42].
